# Multigene Panel Sequencing Identifies a Novel Germline Mutation Profile in Male Breast Cancer Patients

**DOI:** 10.3390/ijms241814348

**Published:** 2023-09-20

**Authors:** Ayman Al Saati, Pierre Vande Perre, Julien Plenecassagnes, Julia Gilhodes, Nils Monselet, Bastien Cabarrou, Norbert Lignon, Thomas Filleron, Dominique Telly, Emilie Perello-Lestrade, Viviane Feillel, Anne Staub, Mathilde Martinez, Edith Chipoulet, Gaëlle Collet, Fabienne Thomas, Laurence Gladieff, Christine Toulas

**Affiliations:** 1 Oncogenetics Laboratory, Oncopole Claudius Regaud, IUCT-Oncopole, Toulouse, France; alsaati.ayman@iuct-oncopole.fr (A.A.S.); telly.dominique@iuct-oncopole.fr (D.T.); lestrade.emilie@iuct-oncopole.fr (E.P.-L.); 2 DIAD, Inserm, Centre de Recherches en Cancérologie de Toulouse, Toulouse, France; thomas.fabienne@iuct-oncopole.fr; 3 Université de Toulouse, Université Toulouse III-Paul Sabatier, Toulouse, France; 4 Bioinformatics Department, Oncopole Claudius Regaud, IUCT-Oncopole, Toulouse, France; plenecassagnes.julien@iuct-oncopole.fr; 5 Biostatistics Department, Oncopole Claudius Regaud, IUCT-Oncopole, Toulouse, France; juliagilhodes@gmail.com (J.G.); monselet.nils@iuct-oncopole.fr (N.M.); cabarrou.bastien@iuct-oncopole.fr (B.C.); filleron.thomas@iuct-oncopole.fr (T.F.); 6 Oncogenetics Department, Oncopole Claudius Regaud, IUCT-Oncopole, Toulouse, France; lignon.norbert@iuct-oncopole.fr (N.L.); feillel.viviane@iuct-oncopole.fr (V.F.); a.staub@ch-montauban.fr (A.S.); chipoulet.edith@iuct-oncopole.fr (E.C.); collet.gaelle@iuct-oncopole.fr (G.C.); gladieff.laurence@iuct-oncopole.fr (L.G.); 7 Oncology Department, ONCORAD Clinique Pasteur, Toulouse, France; mmartinez@clinique-pasteur.com; 8 Pharmacology Department, Oncopole Claudius Regaud, IUCT-Oncopole, Toulouse, France

**Keywords:** male breast cancer predisposition, large multigene panel sequencing, MBC-specific mutated profile, new genes potentially involved in MBC risk

## Abstract

Even though male breast cancer (MBC) risk encompasses both genetic and environmental aetiologies, the primary risk factor is a germline pathogenic variant (PV) or likely pathogenic variant (LPV) in *BRCA2, BRCA1* and/or *PALB2* genes. To identify new potential MBC-specific predisposition genes, we sequenced a panel of 585 carcinogenesis genes in an MBC cohort without *BRCA1/BRCA2/PALB2* PV/LPV. We identified 14 genes carrying rare PVs/LPVs in the MBC population versus noncancer non-Finnish European men, predominantly coding for DNA repair and maintenance of genomic stability proteins. We identified for the first time PVs/LPVs in *PRCC* (pre-mRNA processing), *HOXA9* (transcription regulation), *RECQL4* and *WRN* (maintenance of genomic stability) as well as in genes involved in other cellular processes. To study the specificity of this MBC PV/LPV profile, we examined whether variants in the same genes could be detected in a female breast cancer (FBC) cohort without *BRCA1/BRCA2/PALB2* PV/LPV. Only 5/109 women (4.6%) carried a PV/LPV versus 18/85 men (21.2%) on these genes. FBC did not carry any PV/LPV on 11 of these genes. Although 5.9% of the MBC cohort carried PVs/LPVs in *PALLD* and *ERCC2,* neither of these genes were altered in our FBC cohort. Our data suggest that in addition to *BRCA1/BRCA2/PALB2*, other genes involved in DNA repair/maintenance or genomic stability as well as cell adhesion may form a specific MBC PV/LPV signature.

## 1. Introduction

Male breast cancer (MBC), a rare cancer and a poorly understood malignancy, represents less than 1% of all cancers in men and all breast cancers in Western countries [1], but its incidence is increasing worldwide. A man’s lifetime risk of breast cancer is approximately 1:1000, compared to 1:8 for a woman [2]. On average, MBC cases are diagnosed at an older age than women (67 versus 62 years). The breast cancer prognosis, corrected for age and tumour stage, is similar for men and women, but men are often diagnosed later in life and therefore at a more advanced stage of disease, which is reflected in their lower overall survival rate [3,4]. MBC is often compared to postmenopausal breast cancer in women due to the relatively older age at onset and the high prevalence of estrogen receptor (ER) positivity. Nevertheless, several lines of evidence suggest that MBC tumours differ from the female equivalent. Although genomic approaches are not as developed in MBC as they are in female breast cancer (FBC), several studies have identified sex-specific differences in mutated genes [5] as well as copy-number variations [6] or epigenetic mutations [7]. Given the rarity of the disease, few MBC clinical trials have been conducted to date. The mainstay of MBC treatments, therefore, follows the same recommendations as FBC treatments and includes hormone-, chemo- and radiotherapy as well as targeted therapies such as PARP inhibitors for germline *BRCA1/BRCA2*-mutation carriers. However, the differences between MBC and FBC have led the American Association of Clinical Oncology (ASCO) to recently publish guidelines providing practice recommendations for the specific management of MBC [2].

MBC risk factors encompass both genetic or environmental factors: a personal and/or family history of cancer [8]; ethnicity [9]; a hormonal imbalance [10] such as Klinefelter syndrome [11]; obesity; and exposure to specific toxic substances such as solvents, exhaust gases, petrol and ionizing radiation [12]. Having a first-degree relative with breast cancer increases the risk of MBC (relative risk (RR) = 1.92); the risk increases even more with an affected sister (RR = 2.25), with a dramatic increase observed when the family history includes both an affected mother and an affected sister (RR = 9.73) [11]. To date, the only clearly identified risk factor for MBC is the presence of a germline pathogenic or likely pathogenic variant (PV or LPV, respectively) in the *BRCA2* or *BRCA1* genes. Several studies have investigated the involvement of other genes in the risk of developing MBC, with populations ranging from 6 to 767 patients and panels ranging from 16 to 94 genes, predominantly restricted to genes previously implicated in FBC [13,14,15,16] or even at the exome scale (4600 genes), albeit only including 6 patients [17]. These studies have demonstrated the presence of PVs associated with the risk of MBC in the *PALB2* (OR = 6.6–17.3), *CHEK2* (OR = 3.7), *ATM* and *RAD51D* (OR = 8.6–10.2) genes (see review [18]). A recent study identified three novel MBC susceptibility loci, two mapping to 6q25.1 and one to 11q13.3, which were also associated with FBC risk [19].

In France, all MBC cases are offered genetic counselling and germline genetic testing of cancer predisposition genes. Genetic testing is currently performed on a consensus panel of predisposition genes, initially *BRCA1/BRCA2/PALB2* genes, and now an HBOC (hereditary breast and ovarian cancer) panel including *BRCA1, BRCA2, PALB2, TP53, CDH1, PTEN, RAD51C, RAD51D, MLH1, MSH2, MSH6, PMS2* and *EPCAM* genes. However, these genetic panel analyses only reveal the presence of a PV in approximately 15–20% of high-risk breast and/or ovarian cancer families, and there are currently no specific MBC risk panels. To identify genes potentially involved in MBC risk in an autosomic dominant model, we focused our attention on rare germline variants presented in an MBC population (that tested negative for *BRCA1/BRCA2/PALB2* genes) by investigating a panel of 585 genes. We then compared the germline mutation profile of these genes detected in MBC to that of a population with FBC.

## 2. Results

### 2.1. Patient Characteristics

All patients diagnosed with MBC but testing negative for PV/LPV on *BRCA1/BRCA2/PALB2* genes and referred for an oncogenetics consultation in our institute over four years were included in this study (85 patients). Patients in our cohort were diagnosed with MBC at a median age of 66 years (ranging from 23 to 88 years of age), which is similar to the median age classically described in different works [20,21] (Table 1). MBCs were predominantly invasive ductal carcinoma (80.7%), HR-positive (98.8%) and HER2-negative (96.3%) tumours that were treated by hormone therapy (90.1%). The majority of patients were Caucasian (98.8%), with 1.2% from other ethnic groups. Our cohort presented with 27.1% having first-degree relatives with a history of breast and/or ovarian cancer and 8.2% with an affected second-degree relative. Twenty-five patients were diagnosed with a second cancer: bilateral breast (n = 5), prostate (n = 12) and colon, urinary tract or lung cancers (n = 8). More than half of our MBC patients (58.7%) had a BMI greater than 25, and 15.9% had a BMI greater than 30; furthermore, 35.2% of them had smoked tobacco at some stage during their lives.

### 2.2. Germline Pathogenic and Likely Pathogenic Variants 

**Figure 1:**
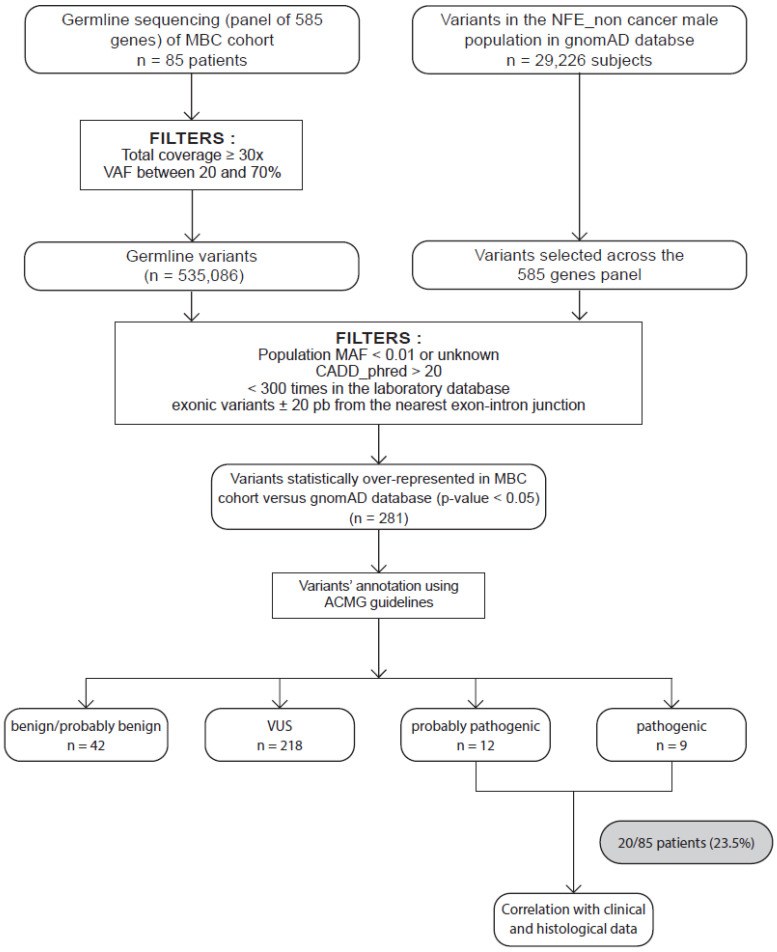
Strategy to identify candidate MBC susceptibility genes. Eighty-five patients with male breast cancer (MBC) were included. Germline variants of a panel of 585 genes were sequenced and aligned to the human genome hg19 before variant calling and annotation. All germline variants were identified and filtered as described in the Materials and Methods section. We obtained a total of 535086 variants. In parallel, we collected and applied the same filters on variants in the GnomAD database restricted to male noncancer NFE and selected across our 585-gene panel. Statistical tests were performed on 495 variants. We then selected the variants preferentially detected in our MBC population (adjusted p-value < 0.05), identifying 281 variants. These variants were then classified as PV, LPV, or variant of uncertain significance (VUS) and likely benign (LBV) or benign (BV) according to the ACMG criteria. We obtained 12 LPVs and 9 PVs. We also performed correlations with clinical and histological characteristics of patients.

The sequencing of our 585-gene panel generated a mean of 6295 variants per patient. Figure 1 describes the filters used to select the variants. Briefly, we selected rare coding variants with a population minor allele frequency from GnomAD (MAF) < 1% and a CADD_phred score > 20 [22,23], and we excluded variants previously detected >300 times from our sequencing databases to avoid introducing recurrent artefact-related variants. The same filters were applied to the GnomAD database v2.1.1 restricted to male noncancer non-Finnish Europeans (NFE). We then selected the variants preferentially detected in our MBC population (adjusted p-value < 0.05), thus identifying 281 variants. The American College of Medical Genetics and Genomics (ACMG) criteria [24] were manually applied to classify 218 variants of unknown significance (VUS; Supplementary Tables S1 and S2) and 21 PVs/LPVs preferentially detected in our MBC population compared to the male NFE (Table 2). Some of these PV/LPV carriers also carried one or several ACMG-classified VUS (up to six different VUS per patient; Supplementary Table S2), including for patient #40 a *PALB2* VUS. According to ACMG recommendations concerning the nonuse of VUS in clinical decision, we restricted our present study to PVs or LPVs. Five of the PV/LPV-carrying genes identified had already been associated with an increased risk of FBC in the case of *BARD1* (*BRCA1*-associated Ring Domain 1) and *MRE11* (*MRE11* homolog, double-strand break repair nuclease); colon cancer in the case of *MUTYH* (mutY DNA glycosylase) when both copies of the gene are mutated; ovarian cancer in the case of *RAD51C* (RAD51 paralog C); or pancreatic cancer and melanoma in the case of *CDKN2A* (Cyclin Dependent Kinase Inhibitor 2A). Men carrying these PVs/LPVs did not report any familial history of colon, pancreatic or ovarian cancer, although we did not have a family history for patient #32, who was adopted (Table 2).

### 2.3. MBC Patients with Germline Variants

A number of MBC patients who had at least one PV/LPV had a personal history of cancer: prostate carcinoma for patients #42 (diagnosed at 72 years), #56 (at 68 years) and #72 (at 68 years), melanoma for patient #41, lymphoma for patient #18 and gallbladder carcinoma for patient #32. Only three MBC patients with a PV/LPV had a first-degree relative with breast or ovarian cancer history: patient #41 was identified with a *RECQL4* PV/LPV and diagnosed with MBC at 77 years of age (his sister was diagnosed with breast cancer at 35 and his brother with pancreatic cancer at 79), patient #83 had a *PALLD* PV/LPV and was diagnosed with MBC at 59 years of age (his mother was diagnosed with breast cancer at 55) and patient #14 was an *ERCC2* PV/LPV carrier and was diagnosed with MBC at 77 years of age (his mother was diagnosed with ovarian cancer at the age of 88).

**Figure 2:**
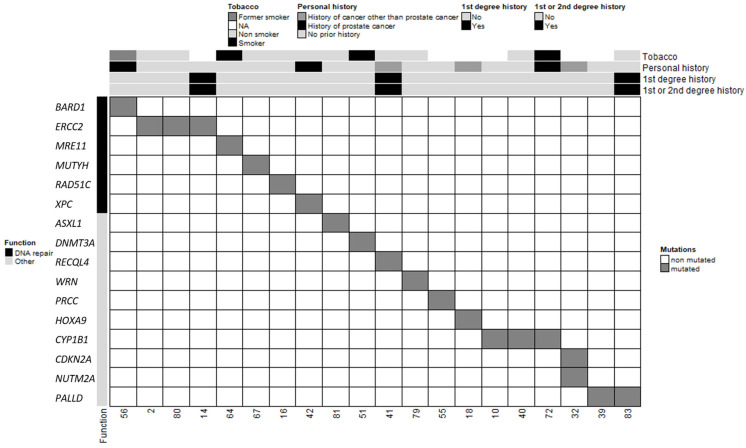
Clinical and molecular characteristics of MBC patients and PVs/LPVs. Each column corresponds to a patient (patient ID specified). The upper section shows the patient’s clinical characteristics (smoking status, personal history of cancer, first-degree relatives with breast and/or ovarian cancer and first- and second-degree relatives with breast and/or ovarian cancer).

In total, 20 patients in our cohort carried at least one PV or LPV in 1 of the 585 genes screened, and 3 patients carried 2 variants (Table 2 and Figure 2). Among the three patients with two distinct PVs/LPVs, patient #32 was notable because his two variants mapped to two distinct genes: *CDKN2A* and *NUTM2A*. Two other MBC patients (patients #2 and #80) carried the same two PVs on the *ERCC2* gene: c.1381C>G and c.2150C>G. The literature describes multiple individuals in whom these two *ERCC2* variants occur in *cis* and as part of a complex allele of compound heterozygous genotypes with other pathogenic alleles and features of xeroderma pigmentosum (XPD; OMIM #278730) and trichothiodystrophy (TTD1; OMIM #601675) [25,26,27]. Our two MBC patients carrying these two *ERCC2* variants did not present any of the characteristics of these diseases. Patient #2 only reported a case of a brain tumour in a paternal aunt diagnosed between 40 and 50 years of age, and patient #80 did not report a family history of cancer.

The other PVs/LPVs detected in our MBC cohort mapped to 13 distinct genes (Figure 2). However, because *DNMT3A* and *ASXL1* often contain somatic mutations in a variety of adult hematologic malignancies [28], we chose to exclude these variants from subsequent analyses. The remaining 11 genes predominantly encode proteins involved in DNA repair (*MUTYH, MRE11, XPC, RAD51C* and *BARD1*), maintenance of genomic stability (*RECQL4, WRN*), pre-mRNA processing (*PRCC*) and transcription regulation (*HOXA9*). These results suggest that the risk of MBC may be mainly associated with DNA repair and maintenance of genomic integrity. Five patients presented with PVs/LPVs in genes that control other cellular pathways such as the *PALLD* gene, which encodes the f-actin organising protein palladin (patients #39 and #83), and the *CYP1B1* gene that encodes the cytochrome P450 family 1 subfamily B member 1 (patients #10, #40 and #72). Segregation of a *PALLD* PV (c.2176C>T, p.Pro726Ser) was first identified in members of a high-risk pancreatic cancer family [29]. More recently, another *PALLD* PV (c.154G>A, p.Asp52Asn) was reported in another high-risk pancreatic cancer family [30]. However, patients #39 and #83 did not have a family history of pancreatic cancer. *CYP1B1* encodes for a key estrogen metabolism enzyme, which metabolises polycyclic aromatic hydrocarbons and 17beta-estradiol [31]. We did not have any information relating to the exposure of patients #10, #40 and #72 to hydrocarbons. The preferential expression of *CYP1B1* PVs/LPVs in our MBC population when compared to NFE males suggests that a constitutive defect in *CYP1B1*-controlled metabolism may increase the risk of MBC in men exposed to these hydrocarbon molecules during their lives.

### 2.4. Clinical and Family Characteristics of MBC PV/LPV Patients

**Figure 3:**
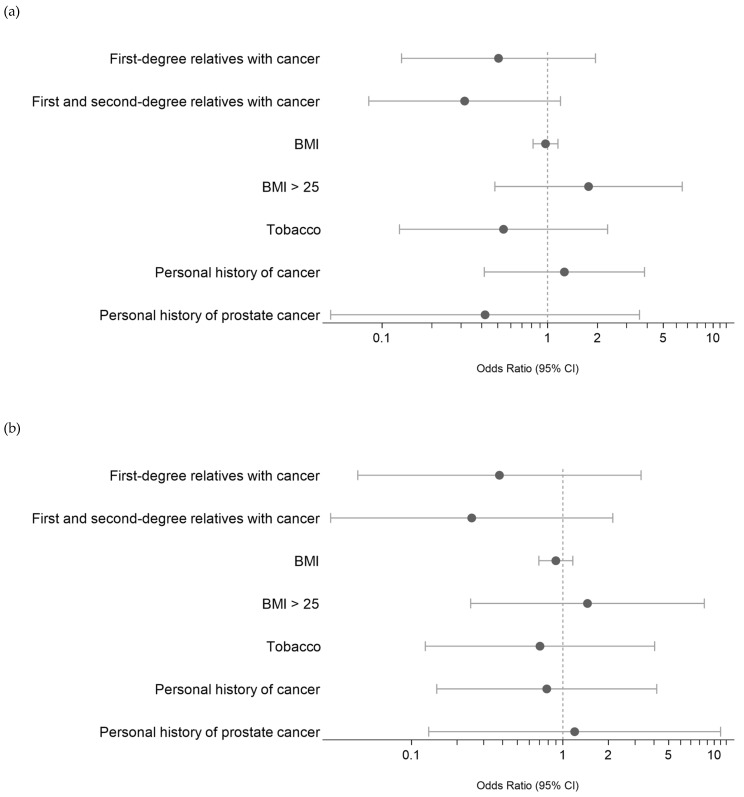
(**a**) Risk evaluation (ODD) of PV/LPV germline variants in MBC based on clinical characteristics. References of categorical values: first- or first- and second-degree relatives with cancer: presence of cancer; personal history of cancer: presence; smoking status = former and current. (**b**) Risk evaluation of PV/LPV germline DNA damage variants in MBC based on clinical characteristics. References of categorical values: first- or first- and second-degree relatives with cancer: presence of cancer; personal history of cancer: presence; smoking status = former and current.

We then attempted to identify any potential associations between these PVs/LPVs (*DNMT3A* and *ASXL1* excluded) and the clinical characteristics of MBC patients, namely, the presence of a first- or second-degree relative with cancer, personal history of cancer, BMI and smoking. As shown in Figure 3A, we did not find any significant association. A subanalysis of the eight MBC patients with PVs/LPVs in DNA repair pathway genes (Figure 3B) also found no significant association.

### 2.5. Study of the 14 MBC PV/LPV-Carrying Genes in a Population of High-risk Breast Cancer Women 

To investigate whether MBC and FBC risk might be at least in part due to different genes, we then examined their mutability in a population of high-risk breast cancer women without *BRCA1/BRCA2/PALB2* PV/LPV detected on the same 585-gene panel analysis. These women (n = 109) had families that fulfilled the criteria admitted for high-risk breast/ovarian cancer families according to the national recommendations; all of them were addressed to the oncogenetics medical consultation of our institute. We examined whether the fourteen MBC PVs/LPVs were also present in high-risk breast cancer women. Similar to our MBC cohort, we selected women diagnosed with invasive intraductal carcinoma (95.4%), HR-positive and HER2-negative tumours (92.7%) and screened the whole coding region of the 14 previously identified genes for any PVs/LPVs. As shown in Table 3, only 5/109 women (4.6%) were identified as having a PV/LPV in these fourteen genes versus 18/85 men (21.2%). Among these genes, 11 did not contain any PVs/LPVs in the FBC population. These genes that were not mutated in the FBC cohort also included genes involved in DNA repair functions (*MUTYH, RECQL4, BARD1, ERCC2* and *XPC*) and other cellular functions (*CDKN2A, HOXA9, NUTM2A, PALLD, PRCC* and *WRN*). Three MBC patients presented one PV/LPV in the *ERCC2* gene and two MBC patients in the *PALLD* gene, but none presented in our FBC population. These results show that the PV/LPV profiles identified in our MBC cohort are not identical to our FBC population.

## 3. Discussion

Our work identified germline PVs/LPVs from a panel of 585 genes in a population of 85 MBC *BRCA1/BRCA2/PALB2*-negative patients. We detected PVs/LPVs in fourteen genes preferentially mutated in our MBC population when compared to a male noncancer NFE population (*DNMT3A* and *ASXL1* excluded). Previous works have tended to identify genes involved in MBC risk based on either more or less restricted panels of genes on large MBC populations or exome analysis of small MBC populations. Bucalo et al. recently screened 1349 MBC cases with a 50-gene panel and reported the involvement of *ATM* and *PALB2* genes in the risk of MBC [16]. Another study examining 94 genes in 102 Greek MBC patients identified PVs in 6 genes: *BRCA2, ATM, BRCA1, CHEK2, PMS2* and *FANCL* [15]. In a cohort of Italian MBC (523 patients) analysed with a panel of 50 cancer-associated genes, *PALB2* and *RAD51D* gene variants were significantly associated with MBC risk [21]. Whole exome sequencing performed on six MBC cases revealed germline variants in *BRCA2, MSH5, DCC, ERBB3, NOTCH3, DIAPH1* and *DNAH11*, but no statistical tests were performed in this study [17]. Our current study showed that our MBC cohort presents a distinct mutation profile when compared to a male noncancer NFE population by preferentially carrying at least one PV/LPV variant in the *CDKN2A, HOXA9, NUTM2A, PALLD, PRCC, RECQL4, WRN, CYP1B1, BARD1, ERCC2, MRE11, MUTYH, RAD51C* or *XPC* genes. Our results are consistent with a previous study identifying *BARD1* and *MUTYH* mutations in MBCs [21]. *BARD1* (*BRCA1*-associated Ring Domain 1), which encodes a nuclear partner of *BRCA1* interacting with the N-terminal region of *BRCA1*, has been identified as a moderate penetrance predisposition gene in FBC [32]. The high allelic frequency of the more common *MUTYH* variants in the general population may explain the concordant *MUTYH* results in our study and the Rizzolo et al. study (c.1105delC in our study (MAF = 0.01% in NFE population); c.494A > G (MAF = 0.25% in NFE population) and c.679C > T (MAF = 0.005% in NFE population) in Rizzolo et al.’s work) [21].

Consistent with what has been described in the literature, our study confirms that PVs/LPVs in MBC patients predominantly map to key DNA repair genes (*ERCC2, MRE11*, *XPC, RAD51C* and *BARD1*) or to genes that safeguard genomic stability (*RECQL4, WRN*). The DNA repair pathways controlled by these genes involve nucleotide excision repair pathways (*ERCC2, XPC*), homologous recombination (HR) repair pathways (*BARD1, RAD51C*) and nonhomologous DNA end joining (NHEJ) repair pathways (*MRE11*). Our data are in line with previously published data [21] which suggest that, as for other cancer predisposition syndromes, some MBCs may be favoured by a constitutive defect in at least one DNA repair pathway. Our work demonstrated that genes involved in pre-mRNA processing (*PRCC*) and transcription regulation (*HOXA9*) are also preferentially mutated in our MBC cohort. A *PRCC* (which codes for the Proline Rich Mitotic Checkpoint Control Factor) and *TFE3* (transcription factor E3) gene translocation was identified in papillary renal carcinoma in 1996 [33]. *HOXA9*, a member of the homeobox gene family that controls gene expression during early development, plays a crucial role in haematopoiesis. Indeed, *HOXA9* dysregulation is necessary for leukemic transformation [34]. *RECQL4* codes for a DNA helicase of the Rec family, which unwinds double-stranded DNA into single-stranded DNA and is a key factor involved in maintaining genomic stability. Homozygous or compound heterozygous germline mutations in the *RECQL4* gene are linked to the recessive syndromes Rapadilino syndrome (RAPADILINO syndrome; OMIM #266280) and Baller–Gerold syndrome (BGS; OMIM #218600). Recent work investigating the cancer risk in a cohort of 123 individuals with heterozygous germline *RECQL4* mutations showed that the prevalence of cancer was not increased in these patients but that patients with type II Rothmund–Thomson syndrome with biallelic REC mutations (including *RECQL4*) were specifically at increased risk of developing lymphomas and osteosarcomas [35]. Another member of the Rec family, *WRN*, is associated with Werner syndrome (WS; OMIM #277700) and increases the risk of thyroid carcinoma, melanoma, breast cancer and meningioma as well as soft tissue and bone sarcomas [36]. None of our MBC patients presented with the characteristic phenotypes of these different syndromes. To the best of our knowledge, no germline PVs have to date been reported in the *PRCC, HOXA9, RECQL4* or *WRN* genes in MBC patients. These results suggest that in addition to the DNA repair pathways typically reported in cancer predisposition syndromes, pathways controlling genomic stability, in particular heterozygous PVs in genes of family members of the Rec helicases, may also be involved in the risk of some MBCs.

Our study also identified PVs/LPVs in four other genes involved in cellular functions independent of DNA repair or genomic integrity functions (*CDKN2A, NUTM2A, PALLD* and *CYP1B1*). *CDKN2A* is a well-established melanoma and pancreatic cancer predisposition gene. Given that the family history of patient #32, who had a germline PV in the *CDKN2A* gene, is unknown, a familial predisposition to these two cancers cannot be ruled out. *CYP1B1* (cytochrome P450 1B1), a member of the cytochrome P450 family, is involved in the metabolism of estrogens and a variety of xenobiotics, such as polycyclic aromatic hydrocarbons, which can be oxidized to active carcinogenic products by *CYP1B1*. Homozygous or compound heterozygous germline mutations in the *CYP1B1* gene are linked to the recessive syndrome primary congenital glaucoma (GLC3A; OMIM #231300). *CYP1B1* polymorphisms have also been found to increase the risk of prostate and breast cancer [37,38]. Three patients in our MBC cohort carried one of the three different PVs/LPVs, two of which were predicted to encode a truncated, potentially inactive CYP1B1 protein. Several studies have, in turn, shown significant associations between MBC risk and estrogen imbalance [10] as well as exposure of men to polycyclic aromatic hydrocarbons [39]. It may be reasonable to extrapolate that the presence of *CYP1B1* gene PVs/LPVs that affect both estrogen and hydrocarbon metabolism might increase the risk of developing MBC, at least in men exposed to hydrocarbons. Interestingly, our study of 85 MBCs did not identify any associations between MBC and a patient or family history of cancer. This may be consistent with MBC predisposition being due to a combination of genetic variants affecting specific metabolic genes and genes involved in the clearance of environmental hydrocarbons.

We also examined whether the PVs/LPVs in the 14 genes of the 18 MBC patients were present at similar frequencies in a cohort of 109 high-risk breast/ovarian cancer women without *BRCA1/BRCA2/PALB2* PV/LPV. It was notable that two genes, *PALLD* and *ERCC2*, presented PVs/LPVs in 5.9% of our MBC population (respectively, 2.4 and 3.5%) but none in our 109 FBC patients. The *PALLD* gene encodes palladin, a protein that regulates actin cytoskeleton organisation as well as cell adhesion and migration/invasion [40]. Germline mutations in *PALLD* have been reported in high-risk pancreatic cancer families [29,30,41]. To date, only one study has reported *PALLD* germline variants in a woman diagnosed with breast cancer at the age of 38 from a high-risk breast/ovarian cancer Chinese family (a total of 389 patients investigated) [42], but the involvement of this variant in breast cancer risk remains to be clearly established. To the best of our knowledge, we are the first study to demonstrate that germline PVs/LPVs mapping to a gene involved in cellular invasion, *PALLD*, are represented at an increased prevalence in an MBC cohort compared with a male noncancer NFE and an FBC population. The second gene, *ERCC2*, encoding an adenosine triphosphate (ATP)-dependent DNA helicase, is frequently mutated in families with increased nasopharyngeal carcinoma risk [43]. A polymorphism in this gene (p.Asp312Asn) is associated with bladder, oesophageal, and gastric cancers but not with breast or prostate cancer [44]. Taken together, we show that 11 out of the 14 genes tested in our MBC cohort (n = 85) contained PVs/LPVs but that these 11 genes were all wild type in the FBC cohort (n = 109). This suggests that in addition to the well-established high-risk breast cancer genes previously described in FBC and MBC, other genes involved in cell adhesion and DNA repair may form part of a specific MBC risk signature. 

To conclude, we have identified germline PVs/LPVs in genes not previously associated with MBC risk, which may suggest a potential specific role of at least some of these genes in male breast cancer. However, our present results do not allow us to firmly associate these genes with MBC risk. To further explore the involvement of these genes in MBC risk, larger MBC population studies will be needed along with FBC cohorts to firmly establish the presence of a specific MBC profile risk. In the meantime, identifying variants in these MBC-specific genes in the clinic may be important for better patient care and for guiding personalized therapy, as evidenced by the success of PARP inhibitors in patients with *BRCA1/BRCA2*-mutated cancers or other defects in homologous recombination DNA repair mechanisms including those reported here.

## 4. Materials and Methods

### 4.1. Patient Cohort

This is a retrospective study of inherited defects in cancer genes in all patients diagnosed with MBC, followed by the oncogenetics department at the IUCT-Oncopole, Toulouse (France), between 2017 and 2020. This study included 85 consecutive MBC patients who tested negative for pathogenic *BRCA1/BRCA2/PALB2* gene variants. Clinical data, tumour characteristics and molecular results were retrospectively reviewed for all MBC patients. Informed consent for using information and biological samples was obtained from all participants. For comparison with FBC and for descriptive purposes only, we selected a population of 109 FBC (without MBC family history) from the last ones referred to our laboratory to perform the same genetic analysis. All these women were *BRCA1/BRCA2/PALB2*-negative and shared the same tumour characteristics as the male population.

### 4.2. NGS Analysis Panel of 585 Genes

DNA was extracted from whole blood with a QIAamp DNA Blood Mini kit (Qiagen, Hilden, Germany), captured using a KAPA library amplification kit (Roche; list of genes available on Supplementary Table S3) and sequenced on an Illumina NextSeq500 sequencer (Illumina, San Diego, CA, USA) according to manufacturers’ instructions. Germline variants were then aligned to the human genome hg19 and called using HaplotypeCaller from GATK 3.3.0 [45] and VarScan 2.3.7 [46]. Only high-confidence variants (total sequencing depth ≥ 30 and variant allele frequency (VAF) between 20% (to avoid any sequencing artefact) and 70% (to avoid homozygous variants)) were reported in this study. These variants have been confirmed by a review of BAM files with Integrative Genomics Viewer (IGV) [47].

### 4.3. Germline Data Analysis

The sequencing data were analysed for the presence of single nucleotide variants and small insertions and deletions (coding variants or variants which were less than 20 bp away from the nearest exon–intron junction). All variants with a frequency of less than 1% in the public GnomAD v2.1.1 database were investigated and then manually classified according to the published ACMG recommendations [24]. Classification criteria included information from curated databases, computer predictions of the effects of mutations on protein function and data from the medical literature. PVs/LPVs including nonsense, frameshift, splice site and missense variants were included. For missense variants, we only selected those with published evidence supporting a pathogenic or likely pathogenic function in the ClinVar [48] or Uniprot databases [49]. Details of the data analysis and interpretation are provided in Figure 1.

### 4.4. Statistical Analysis

Categorical variables are expressed as frequencies and percentages and continuous variables as medians and ranges. Comparisons of individual variant frequencies between MBC and a control male NFE population from the GnomAD database were performed using the Fisher exact test with a Benjamini–Hochberg procedure for multiple testing. Associations between clinical characteristics and the presence of LPV or PV were assessed using a logistic regression model. Odds ratios (ORs) were estimated with a 95% confidence interval (95% CI). All statistical tests were two-sided, and p-values < 0.05 were considered statistically significant. Statistical analyses were conducted using R (v4.0.2) and STATA software (v16) (Stata Corporation, College Station, TX, USA).

## Figures and Tables

**Table 1:** Patient characteristics. Data are presented as n (%, with percentages excluding any missing data) or median (range).

Age at diagnosis (years), median (range)	66 (23–88)
≤65	40 (47.1%)
>65	45 (52.9%)
Personal history of cancer	
No	60 (70.6%)
Yes	25 (29.4%)
Bilateral breast cancer	5 (5.9%)
Prostate cancer	12 (14.1%)
Other cancer	8 (9.4%)
First-degree relatives with breast and/or ovarian cancer	
No	62 (72.9%)
Yes	23 (27.1%)
First- and second-degree relatives with breast and/or ovarian cancer	
No	55 (64.7%)
Yes	30 (35.3%)
Tobacco smoking status	
Never	35 (64.8%)
Former/current	19 (35.2%)
Missing	31
BMI, median (range)	25.6 (17.4–35.2)
≤25	26 (41.3%)
]25-30]	27 (42.8%)
>30	10 (15.9%)
Missing	22
Histology	
Invasive ductal carcinoma	67 (80.7%)
In situ ductal carcinoma	3 (3.6%)
Invasive lobular carcinoma	1 (1.2%)
Invasive mixed type	8 (9.6%)
Other invasive types	4 (4.8%)
Missing	2
Tumour size (mm), median (range)	18.5 (4.0–50.0)
Missing	11
Histological grade	
Grade I	7 (8.4%)
Grade II	55 (66.3%)
Grade III	21 (25.3%)
Missing	2
Lymph node status	
Negative	41 (55.4%)
Positive	33 (44.6%)
Missing	11
ER status	
Negative	1 (1.2%)
Positive	79 (98.8%)
Missing	5
PR status	
Negative	2 (2.5%)
Positive	78 (97.5%)
Missing	5
HER2 status	
Negative	77 (96.3%)
Positive	3 (3.7%)
Missing	5

**Table 2:** Molecular characteristics of PV/LPV germline variants in MBC patients. VAF, variant allele frequency; p-value and ACMG criteria for each variant; MAF, population minor allele frequency from GnomAD.

Function	Patient ID	Gene	NM:cDNA	Protein	VAF (%)	p-Value	ACMG Classification Criteria	MAF
DNA repair	MBC-056	BARD1	NM_000465.3: c.1921C>T	p.Arg641*	30.7	0.013	PVS1 + PM2 + PP5	0.000016
	MBC-002	ERCC2	NM_000400.3: c.1381C>G	p.Leu461Val	47.8	0.0097	PS4 + PS3	0.001203
	MBC-080				52.8			
	MBC-002		NM_000400.3: c.2150C>G	p.Ala717Gly	52.3	0.0097	PS4 + PS3	0.000314
	MBC-080				41.6			
	MBC-014		NM_000400.3: c.2164C>T	p.Arg722Trp	40.9	0.016	PS3 + PM2 + PP3	0.000024
	MBC-064	MRE11	NM_005591.3: c.820_821del	p.Leu274Phefs*16	49.5	0.011	PVS1 + PS4_moderate	0.00002
	MBC-067	MUTYH	NM_001048171.1: c.1105del	p.Ala371Profs*23	52.5	0.018	PVS1 + PM2 + PP5	0.000064
	MBC-016	RAD51C	NM_058216.2: c.414G>C	p.Leu138Phe	47.5	0.011	PS3 + PM2 + PP5	0.000004
	MBC-042	XPC	NM_004628.4: c.1A>G	p.Met1?	40.0	0.018	PVS1 + PM2	0.000007
Chromatin compaction	MBC-081	ASXL1	NM_015338.5: c.1272_1273del	p.Tyr425Glnfs*12	40.6	0.0097	PVS1 + PM2	0.000004
	MBC-051	DNMT3A	NM_175629.2: c.2196dup	p.Glu733*	25.0	0.0097	PVS1 + PM2	0.000004
Cell cycle	MBC-032	CDKN2A	NM_000077.4: c.176T>G	p.Val59Gly	54.6	0.010	PS1 + PS4 + PM2 + PP3	0.000005
Cytochrome P450	MBC-010	CYP1B1	NM_000104.3: c.830del	p.Leu277*	48.7	0.0097	PVS1 + PM2 + PP5	0.000029
	MBC-040		NM_000104.3: c.985G>A	p.Gly329Ser	49.5	0.013	PS3 + PM2	0.000012
	MBC-072		NM_000104.3: c.1064_1076del	p.Arg355Hisfs*69	47.1	0.048	PVS1 + PM2 + PP5	0.000223
Transcription regulation	MBC-018	HOXA9	NM_152739.3: c.802C>T	p.Arg268*	45.2	0.024	PVS1 + PM2	0.000084
DNA helicase	MBC-041	RECQL4	NM_004260.3: c.2547_2548del	p.Phe850Profs*33	44.3	0.011	PVS1 + PM2 + PP5	0.00001
	MBC-079	WRN	NM_000553.5: c.2194C>T	p.Arg732*	50.9	0.0097	PVS1 + PM2 + PP5	0.000016
Other	MBC-039	PALLD	NM_001166108.2: c.814C>T	p.Arg272*	51.1	0.0097	PVS1 + PM2	0.000004
	MBC-083		NM_001166108.2: c.296C>A	p.Ser99*	46.8	0.024	PVS1 + PM2	0.000092
	MBC-055	PRCC	NM_005973.4: c.1138del	p.Gln380Argfs*47	52.4	0.011	PVS1 + PM2	0.000004
	MBC-032	NUTM2A	NM_001099338.1: c.692G>A	p.Trp231*	37.0	0.016	PVS1 + PM2	0.000026

**Table 3:** Comparing the frequencies of PV/LPV-carrying genes in the male and female breast cancer cohorts. The fourteen genes (*DNT3A* and *ASXL1* genes excluded) were examined in a population of 109 women with breast cancer and not carrying PV/LPV on *BRCA1, BRCA2* and *PALB2* genes.

	MBC (%)	FBC (%)
*CYP1B1*	3 (3.5%)	2 (1.8%)
*ERCC2*	3 (3.5%)	0 (0.0%)
*PALLD*	2 (2.4%)	0 (0.0%)
*CDKN2A*	1 (1.2%)	0 (0.0%)
*HOXA9*	1 (1.2%)	0 (0.0%)
*NUTM2A*	1 (1.2%)	0 (0.0%)
*PRCC*	1 (1.2%)	0 (0.0%)
*RECQL4*	1 (1.2%)	0 (0.0%)
*WRN*	1 (1.2%)	0 (0.0%)
*MRE11*	1 (1.2%)	2 (1.8%)
*BARD1*	1 (1.2%)	0 (0.0%)
*MUTYH*	1 (1.2%)	0 (0.0%)
*RAD51C*	1 (1.2%)	1 (0.9%)
*XPC*	1 (1.2%)	0 (0.0%)

## Data Availability

The data presented in this study are available on request from the corresponding author. The data are not publicly available due to the terms of patients’ inform consent.

## Supplementary Materials

The following supporting information can be downloaded at: https://www.mdpi.com/article/10.3390/ijms241814348/s1.
